# A Happy Vision

**Published:** 1920-02-28

**Authors:** 


### A Happy Vision.

A thing of delight to every anxious housekeeper, of value to conditions of healthy existence in the home, but an impossible vision to many, is the all-electric house at the Ideal Home Exhibition. The elimination of fires means the elimination of dust and smoke and labour to a very great degree, but at to-day's prices it would be beyond the reach of the average householder to have these valuable appliances installed now, though it is not so hard a problem in the case of houses yet to be erected. A switch heats water for the bath, another heats the room, another cooks your food, another fans the air, and there are electrical appliances for washing dishes, ironing, and so forth, even to lighting the cigar of the luxurious paterfamilias and his friends. It seems all very idealistic; it would have appeared a mad dream not so many years ago. There is no doubt, however, of its reality now. It is enough to take the heart out of a struggling housewife with no hope of anything but making up and cleaning fires, cooking, and getting hot water, and clearing away the dust and confusion which all such things entail. What a difference it could make to the labours of all the great hospitals and institutions!

The manager of the company which displays the all-electric house looks forward to a time when what now costs £1 for electric service will cost one shilling, and, whether this is rather the height of optimistic commercial propaganda or not, it is the fact that with the proper use and organisation of our power resources electricity should become far cheaper.

The company shows the prices of an all-electric house for four adults in the North of England. No coal or gas has been used during the twelve months. The total current used in the year was on an average of 28.7 units per day. The total cost for the current was given as £32 5s. 9d. for the year. The figures, of course, are only helpful when worked out in relation to the actual cost in the locality of the inquirer. The outfit of the electric house in the Olympia, not counting the duplicate outfit for show purposes, was given as £185.

